# Provider perspectives on service delivery modifications to maintain access to HIV pre‐exposure prophylaxis during the COVID‐19 pandemic: qualitative results from a PrEP implementation project in Kenya

**DOI:** 10.1002/jia2.26055

**Published:** 2023-02-05

**Authors:** Jennifer Velloza, Stephanie D. Roche, Emmah J. Owidi, Elizabeth M. Irungu, Annabell Dollah, Benn Kwach, Nicholas B. Thuo, Jennifer F. Morton, Nelly Mugo, Elizabeth A. Bukusi, Gabrielle O'Malley, Kenneth Ngure, Jared M. Baeten, Kenneth K. Mugwanya, for the Partners Scale‐Up Project Team

**Affiliations:** ^1^ Department of Epidemiology & Biostatistics University of California San Francisco San Francisco California USA; ^2^ Public Health Division Fred Hutchinson Cancer Research Center Seattle Washington USA; ^3^ Partners in Health and Research Development Thika Kenya; ^4^ Jhpiego Nairobi Kenya; ^5^ Washington State University – Global Health Kenya Nairobi Kenya; ^6^ Kenya Medical Research Institute Kisumu Kenya; ^7^ Department of Global Health University of Washington Seattle Washington USA; ^8^ Department of Community Health Jomo Kenyatta University of Agriculture and Technology Nairobi Kenya; ^9^ Gilead Sciences Foster City California USA; ^10^ Department of Medicine University of Washington Seattle Washington USA; ^11^ Department of Epidemiology University of Washington Seattle Washington USA

**Keywords:** adaptation, COVID‐19, HIV, Kenya, pre‐exposure prophylaxis, serodiscordant couples

## Abstract

**Introduction:**

HIV pre‐exposure prophylaxis (PrEP) is an essential prevention strategy being scaled up for priority populations in Kenya, including for HIV serodiscordant couples. The COVID‐19 pandemic posed challenges to PrEP rollout. We conducted a qualitative study of PrEP providers to understand how clinics adjusted PrEP delivery during the COVID‐19 pandemic.

**Methods:**

Since 2017, the Partners Scale‐Up Project has integrated PrEP into 25 HIV clinics in Central and Western Kenya. We conducted qualitative interviews with 40 purposively sampled clinic personnel. We interviewed personnel once during the first pandemic wave (May–Aug 2020) and again after some decline in COVID‐19 rates (Nov–Jan 2021). We analysed data using inductive memo‐writing and summarized data by themes along the PrEP delivery cascade, guided by the Framework for Reporting Adaptation and Modifications (FRAME).

**Results:**

We interviewed 27 clinical officers, five nurses, four health records and information officers, and four counsellors from Central (*n* = 20) and Western (*n* = 20) Kenya. About half (*n* = 19) were female, with a median age of 32 (IQR: 29–34) and 2.3 years of experience delivering PrEP (IQR: 2–3). All participants reported clinic changes in PrEP demand creation and service delivery during the pandemic. Modifications occurred during PrEP implementation and sustainment phases, were partly reactive to the pandemic and also facilitated by interim Ministry of Health guidance on PrEP delivery during COVID, and were made by PrEP delivery teams, clients and clinic managers. Commonly reported modifications included dispensing multiple‐month PrEP refills, intensifying phone‐based client engagement and collaborating with other HIV clinics to ensure that clients with prolonged stays in other regions could continue to access PrEP. Some clinics also adopted practices to streamline visits, such as within clinical‐room PrEP dispensing, pre‐packing PrEP and task‐shifting. Most providers liked these changes and hoped they would continue after the pandemic subsides.

**Conclusions:**

COVID‐19 served as a catalyst for PrEP delivery innovations in Kenya. HIV clinics successfully and rapidly adapted their PrEP demand creation, refill and retention strategies to promote PrEP uptake and effective use. These modified implementation strategies highlight opportunities to streamline the delivery of PrEP, as well as other HIV and chronic care services, and strengthen engagement with populations post‐pandemic.

## 1 INTRODUCTION

Daily oral tenofovir‐based pre‐exposure prophylaxis (PrEP) is an effective HIV prevention intervention that can reduce population‐level HIV incidence if delivered with sufficient coverage [1, 2]. The World Health Organization has recommended its use [3, 4]. Kenya has the fourth largest HIV epidemic in the world and has implemented a number of innovative oral PrEP delivery models for priority populations (e.g. PrEP provision within antenatal care services or in pharmacies) [5, 6]. Oral PrEP has been integrated into clinical settings throughout the country [6, 7]. By August 2022, Kenya had one of the largest PrEP programmes in Africa with over 250,000 individuals having initiated oral PrEP [8]. A number of implementation projects have also been conducted throughout Kenya to catalyse PrEP service delivery for key populations, including the Partners Scale‐Up Project (PSUP), which was launched in 25 high‐volume PrEP clinics in Central and Western Kenya in 2017 and initiated 4898 people in HIV serodiscordant couples on PrEP [9].

In Kenya, prior to the coronavirus (COVID‐19) pandemic, PrEP was predominantly delivered via public HIV clinics, where clients seeking to initiate and/or continue PrEP received “core components” of PrEP delivery: PrEP demand creation (often done by community health workers via health talks outside of clinics or in clinic waiting bays, or with PrEP counsellors or nurses in clinics through partner notification services and contact tracing); HIV risk assessment and testing with an HIV testing services counsellor; medical eligibility for PrEP assessed by a clinical officer or nurse; and PrEP dispensation at the clinic's pharmacy by a pharmacist. Clients seeking to continue PrEP typically returned to the clinic monthly for PrEP refills and, on a quarterly basis, for follow‐up HIV testing [6].

The first COVID‐19 case was reported in Kenya on 13 March 2020 and the pandemic resulted in 284,150 cases and 5364 deaths through December 2021 [10]. Early in the pandemic, Kenya implemented nationwide stay‐at‐home orders and curfews, but publicly funded clinics largely remained open to continue serving clients, including those seeking HIV services. The Kenyan Ministry of Health released interim guidance on 24 March 2020, with suggested modifications to HIV service delivery during the pandemic; however, the guidance focused largely on antiretroviral therapy (ART) provision for people living with HIV. PrEP providers tailored these recommendations and adapted PrEP implementation strategies to fit the needs of clients during the pandemic and innovate efficient strategies for the provision of essential services. Using the Framework for Reporting Adaptation and Modifications (FRAME)—an implementation science framework for documenting programme modifications—we analysed longitudinal qualitative data from interviews with Kenyan PrEP providers collected as part of ongoing PrEP rollout efforts to identify adaptations made by providers to PrEP service delivery in HIV clinics during the COVID‐19 pandemic [11].

## 2 METHODS

### 2.1 Study background and data collection

Between 2017 and 2021, PSUP, in collaboration with the Kenya Ministry of Health, supported the national scale‐up of PrEP in Kenya by providing modular staff training and intensive technical assistance (TA), initially to 25 HIV care clinics and eventually to an additional 75 “mentor clinics” (which were HIV clinics that received the PSUP staff training but no additional TA) [12]. This sub‐study focuses on 40 mentor clinics in Western and Central Kenya to which PSUP staff provided clinic‐based training only. Clinics were purposively sampled to obtain a mix in urban, peri‐urban and rural areas.

PSUP technical assistants approached adult (age 18+) healthcare providers involved in PrEP delivery who spoke either English or Swahili for potential interviews. Interested individuals were subsequently contacted by a research assistant (RA) via phone to schedule an interview. RAs conducted interviews with one participant per clinic facility. All three RAs were trained Kenyan qualitative researchers who had no prior relationship with the interviewees. From August 2020 to January 2021, RAs conducted two one‐on‐one interviews with each participant, approximately 6 months apart. This longitudinal qualitative data collection enabled us to evaluate changes around PrEP delivery modifications during different points in the COVID‐19 pandemic. “First wave” interviews took place from May to Aug 2020 (during the height of the COVID‐19 pandemic in Kenya, when up to 5000 cases were reported per week), and “second wave” interviews took place after some decline in COVID‐19 rates (Nov—Jan 2021, when a maximum of 1000 cases were reported per week) [13]. If a participant was unavailable to participate in a second interview, then another eligible individual from the same clinic was selected for the interview instead, as was feasible given time constraints, clinic schedules and staff turnover. We did not select these “replacement” interviewees to be from the same provider cadre or clinic role as the first wave interviewee at the clinic. During the first wave of interviews, Kenya had implemented nationwide stay‐at‐home orders and nighttime curfews, and although publicly funded clinics largely remained open, reduced open hours, roadblocks and increases in transportation costs presented barriers to clinic access. By the time of the second wave interviews, stay‐at‐home orders and curfews had ceased, and travel within and between regions was more open. Interviews were conducted via phone or in person in a private space within the clinic (while observing COVID‐19 prevention measures) using a semi‐structured interview guide.

Interviews were informed by the FRAME [11]. The FRAME encourages researchers to document and classify modifications around a number of key questions including: *when did the modifications occur (e.g. during implementation, scale‐up, maintenance or sustainment)?*; *were the modifications planned or unplanned?*; *what was modified (e.g. content, context, training, implementation activities)?*; *were modifications made to format, setting, personnel or population?*; *who was involved in the decision to modify (e.g. politician, programme leader)?*; and *what was the nature of the modification (e.g. tailoring, adding elements, removing elements)?* The interview guide solicited information on the following domains: role in PrEP delivery; the impact of the COVID‐19 pandemic on PrEP uptake, adherence and persistence; and adaptations made to clinic operations and PrEP delivery in response to the pandemic, including the rationale for the change, observed outcome and duration (File S1). All interviews lasted approximately 1 hour and were recorded, transcribed and translated. Following each interview, the interviewer summarized key points in a debriefing report for internal use during the analysis process.

### 2.2 Data analysis

We analysed data inductively using conventional content analysis [14]. Authors JV, EO and SR drafted analytic memos that summarized key themes from the first and second wave interviews and their accompanying debrief reports. Where applicable, they noted similarities and differences across sites and updates since the first wave interview (e.g. whether adaptations reported in the first wave interview had been maintained by the second). Memos were written independently but reviewed by at least two authors (JV, EO and SR). Once memos were complete, the authors reread all memos in their entirety and summarized findings in matrix format to compare clinics with respect to the nature of the adaptations and the duration of adaptation implementation during the COVID pandemic.

### 2.3 Data availability and ethical statement

The data that support the findings of this study are available from the corresponding author upon reasonable request. The institutional review boards of the University of Washington and the Kenya Medical Research Institute approved this study. All participants provided written informed consent.

## 3 RESULTS

### 3.1 Participant characteristics and summary of PrEP delivery modifications

We interviewed 40 individuals involved in PrEP delivery during our first‐round interviews, including 27 clinical officers, five nurses, four health records and information officers, and four counsellors (Table 1). Half were from Central Kenya (*n* = 20) and half were from Western Kenya (*n* = 20). Participants had about 2 years of prior experience delivering PrEP at the time of their first interview (median: 2.3 years; IQR: 2.0–3.0). First‐round interviews were conducted with all 40 participants and second‐round interviews were conducted with 29 (72.5%). Of the 29 second‐round interviewees, two were new participants and 27 were participants who also completed a first‐round interview. Although we contacted 40 participants for the second‐round interviews, 11 clinics (five in Central Kenya and six in Western Kenya) were unable to identify available interviewees during the period.

**Table 1:** Participant demographics from Western and Central Kenya, May 2020–January 2021 (*n* = 40)

Characteristic	Frequency^a^
Region	
Western Kenya	20 (50.0%)
Central Kenya	20 (50.0%)
Gender	
Male	21 (52.5%)
Female	19 (47.5%)
Age, years	32 (29–34)
Role	
Clinical officer	28 (70.0%)
Counsellor	4 (10.0%)
Nurse	5 (12.5%)
Other^b^	3 (7.5%)
Training level	
University degree	3 (7.5%)
College 2‐year diploma	35 (87.5%)
Certification	2 (5.0%)
Years working in the clinic	4 (3–5)
Years delivering PrEP	2.3 (2–3)

Abbreviation: PrEP, pre‐exposure prophylaxis.

^a^ Frequencies are provided in *N* (%) for categorical data or median (interquartile range) for continuous data.

^b^ Other includes staff involved in clinic administration or records information officers.

Clinic modifications in PrEP delivery were reported by all 40 participants interviewed in the first wave of data collection across multiple stages of the PrEP delivery cascade: clinic infrastructure and staffing; PrEP demand creation; PrEP initiation; PrEP refill visits; and PrEP retention activities to support persistence (Table 2). Based on the FRAME, we determined that modifications occurred during the PrEP implementation and sustainment phases. Modifications were somewhat unplanned and reactive to the COVID‐19 pandemic, but were also informed and supported by the Kenyan Ministry of Health's interim guidance on HIV service delivery for people living with HIV (released on 24 March 2020). That guidance described changes related to the length of antiretroviral medication refills and remote service provision, that PrEP providers tailored for PrEP delivery. Clinic staff participated in the decision to modify, although modifications were also informed by PrEP recipients who provided feedback about how well the changes worked for them and whether they would like to see changes maintained after the initial height of the COVID pandemic. These were fidelity‐consistent modifications, in that they preserved the core components of PrEP delivery in form and function.

**Table 2:** Modifications in PrEP delivery in Central and Western Kenya reported during the first wave of the COVID‐19 pandemic (May–Aug 2020) and after some decline in COVID (Nov–Jan 2021)

PrEP delivery cascade component	Practice prior to the COVID‐19 pandemic	Changes reported during the first interview	Maintenance of changes in subsequent interview	Representative quotations
Clinic infrastructure and staffing	Most clinics offered PrEP services in the HIV care and treatment area, with PrEP delivered primarily by ART providers	Two clinics became COVID‐19 isolation centres and relocated PrEP delivery to other areas of the clinic; hours reduced to accommodate curfew times; some reduced staffing, created staggered staffing shifts or used task‐shifting (e.g. clinics have one clinician to complete both adherence counselling and clinical review, rather than two separate providers) to decongest clinics which contributed to provider burnout; PrEP was integrated into other clinic services	Clinics resumed normal operating hours; about half the clinics stopped conducting or enforcing temperature checks; staff have been encouraged to take leave to minimize burnout; several clinics stopped with staggered staff shifts	“So maybe the strain is…you have an elderly person who is a staff, and you feel they are insecure with their age and their health. Then there is COVID, and you have a positive staff, so they won't come in. You have to cover in for them and there is no substitution, you won't get extra workforce.” “We created teams—the CO (clinical officer), one peer educator and a counsellor. Those are three; that is one team. So, if maybe they report today; another team reports the next day, to at least reduce the number of staff in a room.” “Let's say when I have a client and maybe a VCT counsellor's room doesn't have a client, I would go and use his room. You just find space.”
PrEP demand creation	Health talks in waiting bays; community outreach activities; contact tracing and partner notification	Most activities suspended or scaled back; intensified phone‐based contact tracing of individuals newly diagnosed with HIV and partner notification services; increased screening at other clinic departments; passive recruitment with PrEP fliers in clinic waiting rooms; alerting other clinics of PrEP availability at the clinic so they can refer their HIV‐uninfected clients	Several clinics reported resuming health talks and community mobilization but at a social distance and with fewer attendees; continued reliance on partner notification services and one‐on‐one recruitment of partners of clients living with HIV	“We used to go looking for the client to give health talks. That's how we used to get new clients. Staff used to go to bars at night. Most of the clients who would be brought in were being got from bars…But during COVID‐19, they could not go.” “You see, that kind of outreach couldn't happen with COVID. Coz that's like now we are going to the crowd.” “We have had a discussion with our HTS counsellors and we have informed them that they need to disseminate this information to the public that if you have an HIV‐positive partner, then you are supposed to be enrolled in PrEP to prevent you from getting infected. So this has actually gone a long way in convincing the public by accepting this information and therefore we have seen more and more people coming into PrEP.” “We are able to do the PNS (Partner Notification Service) for all the clients so that we can be able to get the status of their partners and if it is a discordant couple, we can enroll them for PrEP. But then getting clients to give you the contacts of their partners is an issue.”
PrEP initiation	Most clinics dispensed 1‐month refills to new initiators and required they return for HIV testing at month 1	Some clinics switched to dispensing 2‐ or 3‐month refills to new initiators and requested they return for HIV testing at month 1; less time spent counselling clients during PrEP initiation and some laboratory procedures were skipped	Most clinics resumed 1‐month PrEP dispensing at enrolment with standard laboratory testing procedures	“The one [new initiator] we gave a TCA of 2 months, you know, he was even very happy. He told us, ‘I thought I would be coming back every month. You did a good thing to give me for 2 months. Next time why don't you give me for 3 months, and I will be taking my drugs well?’” “Okay, we were not taking the weight and heights of the clients. Sometimes you just ask—because there are those tests that are supposed to be done. Mostly we are not [conducting] the LFTs (Liver Function Tests).” “What we tried to consider was the time to have a client one on one. We tried to minimize that length of time… When doing enrollment, I enroll faster and then other things I take the follow up phone and then I call the client to elaborate further about the PrEP to minimize that contact. You find a client has come. I may talk briefly about PrEP. I may touch on the major things like how to take it. I may define the PrEP in a few words then I tell the client that I will elaborate further. The client may not want to stay there for long, so he/she may request for phone contact then we communicate further.”
PrEP refill visits	Most clinics dispensed 1‐month refills to continuing clients	Most switched to dispensing 3‐month refills to continuing clients with good adherence; a few clinics began dispensing PrEP within clinical rooms, pre‐packing PrEP and/or using task‐shifting so that clients could see fewer providers	Most clinics maintained 3‐month refills, particularly to long‐term PrEP clients with good adherence; clinics continued with pre‐packing PrEP as drug supply allowed; clinic flow generally resumed to pre‐COVID duties	“Longer [refills] is convenient to us and to the clients. One thing, is we do not have many clients…because if it is like every month refills, you know there will be many clients. So we reduce that burden and also we reduce the congestion at the facility considering the COVID rules. And then another thing, the clients are happy they can do other things rather than come to the facility every month to take their PrEP drugs.” “I feel longer refills should continue for clients who are competent on PrEP.”
PrEP retention	Some clinics did appointment reminders and followed up with no‐shows by phone	Most clinics have adopted or intensified appointment reminders and follow‐up calls for no‐shows; many temporarily transferred the care of clients (e.g. clients on lockdown in another region) to other clinics or received such transfer clients	Clinics maintained phone calls as an approach to promote retention; several clinics conducted “audits” to determine whether clients who missed visits were still interested in PrEP at the clinic	“The defaulter tracing: if they were supposed to come on a given date and they do not show up, we call them on the appointment date and remind them. If we don't get them or if we get them over the phone, we plan with them when they are supposed to come. Some would say they are having extra pills, so we extend the days depending on the remaining pills. And the ones who are saying maybe they will not make it because they don't have a day off from their workplace, we plan with them who to pick up the drugs maybe from the pharmacy if there is no need of coming to the clinic.” “If somebody is not coming, maybe like initially when the pandemic was real, we could call and somebody could tell you, ‘I am locked down maybe in Mombasa’. So, we would just advise [them] to go to the next facility. Go with your card, tell them you have been on PrEP for this long, the reason as to why you are on PrEP so that they give you a date maybe, they refill for one month then you send us that date. So at least we are sure, this one has not come to the facility but he or she is on drugs somewhere.”

Abbreviations: ART, antiretroviral therapy; COVID‐19, coronavirus; HTS, HIV testing services; PrEP, pre‐exposure prophylaxis; TCA, medication refill; VCT, voluntary counselling and testing.

### 3.2 Clinic operations and staffing

Prior to the pandemic, clinics offered PrEP services in their HIV care and treatment areas and PrEP was primarily delivered by HIV treatment providers who also provided ART to clients living with HIV. The onset of the pandemic brought modifications to the clinic setting where PrEP was delivered, the overall clinic format of PrEP delivery and the personnel who provided PrEP.

Setting modifications included refinements to clinic flow to balance the need for client–provider interaction while mitigating the risk of transmitting COVID‐19. Clinics created PrEP dispensation “windows” (i.e. scheduled times to pick up PrEP refills) to reduce the number of clients in the clinic. They also marked chairs in waiting bays to ensure social distancing and moved triage sections (where vital signs are taken) to different parts of the clinic. Clinic rooms became “flexible spaces” (rather than being assigned to a specific provider) for providers to rotate into as clients needed which helped to decongest the waiting room. Operating hours were reduced (generally from a closing time of 5 PM to 2 or 3 PM) to accommodate COVID‐19 curfews, and all clinics introduced temperature checks and handwashing stations at the entrance gate or in the waiting room. By the second interview, most reported that they no longer routinely enforced temperature checks, handwashing, mask‐wearing and/or social distancing. Clinics also reported difficulty with continuing funding for thermometer batteries, sanitizer or masks. Clinic operating hours resumed to their pre‐pandemic times.

To increase PrEP reach and persistence, clinics implemented format modifications. A total of seven clinics in Western Kenya reported pre‐packaging PrEP (done by peer educators) to reduce client time spent in the clinic. Rather than delivering PrEP as a stand‐alone service, six clinics integrated PrEP into other services (e.g. family planning and outpatient services), which helped reduce the number of interactions between different staff members and clients (thus mitigating the risk of COVID‐19 transmission), improved clinic efficiency (e.g. less queuing for clients at separate points) and reduced stigma related to seeking PrEP. Themes around PrEP service integration and clinic flow efficiency did not differ by the department where PrEP was integrated (e.g. family planning vs. outpatient department). In the second interview, clinics reported maintaining this integrated service delivery:

“Another change was the integration of services. That one is still there because if we set a clinic that is now only focusing on PrEP, then we are stigmatizing. So to avoid the usual stigmatization [of getting PrEP] and to make people used to PrEP, the integration of services is ongoing.” *(Nurse, second wave interview, ID #9, Western Kenya)*

Personnel changes included reducing the number of staff in the clinic at a given time to decongest clinics and minimize COVID‐19 transmission risk. Some clinics (*N* = 7) created staff “teams,” where half the staff would work in a team on alternate days. In addition, nine clinics consolidated staffing roles to compensate for staff shortages. Clients would see 2–3 providers during their visit for multiple services, instead of 5–6 providers for the same number of services, and would see non‐clinician providers as appropriate. This included peer educators who received PrEP training and were compensated for their services:

“[At the beginning of the pandemic,] we were in a situation where we were only two clinicians at a given time in the [clinic]. So once a PrEP client came…we sought the help of the peer educators, and they were able to assist us with the stable clients on PrEP. They would see those clients, and the clinician only had to come in for the clients that needed clinical care.” *(Clinical officer, second wave interview, ID #7 Western Kenya)*

Although task‐shifting from higher‐ to lower‐level cadres helped reduce clinic wait time and overcrowding in the waiting room, it created “burnout” among clinic staff who found themselves performing more than their usual duties to accommodate patient needs. Clinic staff also felt time pressure to see clients quickly with the staggered shifts approach, which reportedly reduced the amount and depth of counselling clients received. Because of these challenges with burnout and time pressure, these modifications generally did not persist: by the second interview, almost all clinics stopped with the staggered shifts and some had ceased the task‐shifting practices.

### 3.3 PrEP demand creation

Prior to the pandemic, PrEP demand creation was largely done through in‐person events, including health talks in clinic waiting rooms, community outreach and community mass testing to identify individuals potentially eligible for PrEP. Clinics also conducted HIV testing contact tracing for clients living with HIV to identify HIV‐negative partners and offered partner notification services. The onset of the COVID‐19 pandemic brought modifications to the format of demand creation activities, with clinics leaning more heavily on remote and small‐scale forms of demand creation like contact tracing and partner notification without community outreach and mass HIV testing events. During the pandemic, clinics in both regions sought new PrEP users primarily through partners living with HIV. They conducted phone‐based contact tracing of individuals newly diagnosed with HIV and those with high viral load. They also worked with partner notification services to invite clients living with HIV to come into the clinic with their partners for joint HIV testing:

“Okay what has changed is how we are getting new clients. We have looked on partner notification service mostly. If we get a new [ART] client, we try to find out if they have partners and that's where we get new clients.” *(Counsellor, first wave interview, ID #12, Central Kenya)*

A total of eight clinics called other nearby health facilities to inform them that they provide PrEP and would welcome referrals of any HIV‐negative clients. In addition, clinics conducted passive recruitment through PrEP posters in clinic waiting rooms. These modifications in PrEP demand creation and recruitment efforts were largely maintained by the second interview.

Clinics also modified the setting of demand creation activities, with PrEP recruitment taking place throughout the clinic. They conducted increased PrEP screening within other clinic departments where patients were seeking non‐HIV‐related services. They held health talks with clients in the waiting areas, including at voluntary counselling and testing points. However, these activities were generally low yield given that clinics were seeing few patients during this time and patients had to social distance:

“So during COVID, we didn't have many health talks. You know for health talks, you talk to a crowd of people that are there, like 10 or more. But during COVID, we didn't have such crowds…the [clinic] was trying to limit people to not be more than 5 [people in the waiting area at a time].” *(Nurse, first wave interview, ID #18, Central Kenya)*

As the COVID‐19 pandemic continued, clinics reported resuming larger, socially distanced health talks in the clinics, particularly for clients seeking HIV testing and treatment services, to reach a greater number of potential PrEP clients than their limited demand‐creation activities during the first COVID wave. A few clinics also resumed community mobilization and outreach events.

### 3.4 PrEP initiation

Kenyan Ministry of Health guidelines specify that a 1‐month PrEP supply should be dispensed at initiation and that clients should return for HIV testing and PrEP counselling after a month. PrEP initiation visits also include a readiness assessment and in‐depth counselling to discuss PrEP efficacy and pill‐taking strategies. Clinics were following these guidelines prior to the COVID‐19 pandemic and valued the opportunity to check in with PrEP clients shortly after they started PrEP to see if they needed any additional support.

PrEP initiation procedures were modified during the pandemic to include lengthening PrEP refills and shortening counselling procedures. The majority of clinics continued with a 1‐month PrEP dispensation at initiation to incentivize repeat HIV testing after a month. However, several clinics reported dispensing 2‐ or 3‐month PrEP supplies to new PrEP initiators to minimize COVID infection risk, lower staff burden and enable clients who could not afford to return to the clinic to stay on PrEP given the higher transport fares:

“Now because of COVID, we are giving longer refills. For a client who has not had any recent exposures, we just give drugs for three months and then encourage them to come back in case they are having side effects. We are just initiating and giving longer refills.” *(Counsellor, first wave interview, ID #8, Western Kenya)*

While one clinic also allowed new PrEP initiators to return after 3 months for HIV testing, all other clinics still requested that new PrEP initiators return for HIV testing and adherence counselling after 1 month as was the standard practice prior to the COVID‐19 pandemic. However, clinics reported spending less time counselling clients during PrEP initiation and skipping medical procedures (e.g. weight assessment) to expedite appointments. By the second interview, all clinics were delivering a 1‐month PrEP supply to new PrEP initiators and conducting standard‐of‐care counselling and medical procedures at PrEP initiation. This resumption of standard PrEP initiation procedures allowed them to more closely follow new initiators and conduct a full medical assessment at initiation to avoid adverse events as much as possible.

### 3.5 PrEP refill visits and retention activities

Prior to the COVID‐19 pandemic, clinics in both regions followed the Kenyan Ministry of Health guidance to dispense 1‐month PrEP refills to continuing clients. During the COVID‐19 pandemic, almost all clinics lengthened PrEP refills to 3 months (with one clinic extending PrEP refills to 6 months). These extended refills were generally only for clients who reported good PrEP adherence. Some staff expressed hesitations about providing longer refills because they were concerned about losing clients or being unable to counsel clients who were less adherent to PrEP with this longer refill schedule. However, staff generally viewed this as a positive change because it decongested the clinic and allowed them to focus resources on clients who needed more attention:

“Longer refills has also helped us. It gives us time to really take time to patients who need help. Like those who are stable [i.e., clients who report good adherence], we give them long refills, leaving behind those patients who really need to be talked to. For example, we get somebody on PrEP and has a lot of issues…So when we give those who are stable long refills, you remain with this patient who really needs help.” *(Clinical officer, first wave interview, ID #14, Western Kenya)*

Clients also appreciated the longer refill schedule because it reduced fears around COVID, as well as the stigma and inconvenience of picking up PrEP refills on a monthly basis. About half the clinics maintained the 3‐month refill schedule for continuing PrEP clients at the second wave interview, although only for those longer‐term PrEP clients who were able to store and take PrEP well:

“For clients that are adherent, [we could] consider a differentiated model with longer refills. Maybe NASCOP [The National AIDS & STI Control Program] can come up with something to categorize “stable on PrEP” and “non‐stable”. If somebody falls under “stable,” they now get the multi‐month dispensing.” *(Clinical officer, second wave interview, ID #10, Central Kenya)*

Prior to the COVID‐19 pandemic, PrEP retention efforts largely included appointment reminder messages and phone follow‐up for clients who missed their clinic visits. The format of these retention efforts was maintained throughout the pandemic. Clinics also intensified their appointment reminder practices (e.g. more frequent phone calls) in response to increased defaulting rates during the pandemic due to clients’ fears of returning to the clinic and contracting COVID‐19.

## 4 DISCUSSION

In this qualitative study of PrEP service delivery adaptations implemented by Kenyan PrEP clinics in response to the COVID‐19 pandemic, participants described modifications to clinic infrastructure and PrEP demand creation, initiation, refills and retention activities. These were primarily context modifications made to the format, setting, and personnel involved and were reported in both Central and Western Kenya. Most clinic staff felt that the modifications successfully enabled clients to continue accessing PrEP during the first months of the pandemic. Moreover, many modifications were maintained at the second interview (e.g. longer PrEP refills for existing PrEP clients, pre‐packaged PrEP and phone‐based reminders) because they improved clinic efficiency and/or were preferred by PrEP clients. Providers also reported that PrEP clients generally liked the modifications, particularly those that improved efficiency in the clinic (e.g. pre‐packaged PrEP and multi‐month PrEP dispensing) and reduced PrEP‐related stigma (e.g. integrated delivery that combined PrEP with other healthcare services), and that these modifications supported PrEP persistence. Results highlight opportunities to streamline PrEP during subsequent waves of the COVID‐19 pandemic and post‐pandemic. Our work is focused on PrEP delivery modifications for HIV serodiscordant couples in Kenya, and results must be interpreted in the context of the PSUP which sought to promote PrEP implementation in Central and Western Kenya. Findings could be generalized to other populations with long‐term PrEP needs (e.g. for whom multi‐month PrEP dispensing may be ideal) and contexts where it may be difficult to attend clinic visits regularly for PrEP alone. Future research is needed to understand client perspectives around PrEP delivery modifications during the COVID pandemic and beyond.

While our work is unique in its focus on PrEP delivery modifications at clinics in Kenya that primarily serve HIV serodiscordant couples, it is relevant to the emerging body of literature related to COVID‐19 service delivery interruptions. Studies in Southern Africa have reported substantial increases in missed PrEP refills during the pandemic, especially during periods of travel restrictions [15]. Mathematical modelling studies have estimated that HIV incidence rates would increase during the COVID‐19 pandemic due to reduced HIV testing and PrEP initiations and adherence [16, 17, 18]. Much like we found in our qualitative study, health systems studies synthesizing early findings from the COVID‐19 pandemic and lessons learned from Ebola outbreaks in West Africa suggest that flexible health systems infrastructure and community‐based engagement with target populations are important approaches to strengthen pandemic responsiveness and health system resilience [19]. In addition, the World Health Organization and PEPFAR have recently recommended modifications to HIV service delivery in line with those employed in Kenya, including decentralized services, extended refills, and innovative recruitment and retention approaches to enable PrEP delivery despite pandemic‐related disruptions [20, 21, 22]. Limited data are available on the impact of such approaches, but two studies of modified ART delivery for persons living with HIV in Africa and Asia found that extending ART refill duration, task‐shifting, phone check‐ins with staff and community‐based ART delivery significantly increased retention and were acceptable for patients and staff [23, 24].

Our longitudinal qualitative data allowed for the evaluation of changes around PrEP delivery modifications during different points in the COVID‐19 pandemic. Initial alarm about the pandemic and lockdowns contributed to sizeable changes to keep clinic doors open and protect staff health. During the time between interviews, COVID‐19 rates declined throughout Kenya, clients and staff became more accustomed to life during the pandemic, and lockdowns and curfews ceased. This provided us with a unique opportunity to explore which initial PrEP modifications were sustained. Prior to the COVID‐19 pandemic, several clinics had already begun modifying how they deliver PrEP, including the adoption of multi‐month PrEP dispensing, phone calls for participants who missed visits and “fast‐tracking” stable PrEP clients (i.e. sending them directly to a clinical room without having to queue) [25], and it is possible that these clinics would have continued with these modifications in the absence of the COVID‐19 pandemic. Yet, our study found that, for some clinics, the pandemic instigated and cemented these changes. Importantly, not all COVID‐related modifications to PrEP delivery were maintained at the time of the second interview and some were perceived to be harmful by providers, including very long PrEP refills for all clients (e.g. 6‐monthly PrEP dispensing irrespective of PrEP adherence) which reduced opportunities for contact with those who may have been having difficulty taking or storing their PrEP. These findings are also an important indication of provider acceptability and feasibility around these modifications and the need to balance client contact and monitoring with reduced burden of attending clinic visits.

Our findings on the types of PrEP delivery modifications, the continued use of several modifications at the second interview, and their feasibility and acceptability to providers highlight opportunities for PrEP programmes to facilitate PrEP initiation, adherence and persistence even after the COVID‐19 pandemic ends. Providers relied on partner notification services to identify potential PrEP clients during the initial phases of the pandemic and continued recruiting clients through this avenue at the second interview. Other studies conducted in Kenya before the pandemic found that the integration of partner notification services with HIV care can successfully improve PrEP implementation and represents a key opportunity to reach at‐risk individuals [26, 27]. While clinics reported stopping community‐based recruitment activities at the time of the first interview, by the second interview, these had mostly resumed, thus highlighting the importance of community sensitization as an approach to reduce PrEP stigma, engage community leaders and improve PrEP awareness for potential clients [28, 29]. Modifications to PrEP refills, including multi‐month PrEP dispensing for stable PrEP clients and pre‐packaged PrEP, were maintained through the second interview and are beneficial both to clients (due to reduced stigma from being seen at the clinic, reduced transportation costs and an appreciation for tailored person‐centred care) and staff (due to improved clinic flow and increased efficiency to spend time with clients who need more support). This is consistent with findings from Zimbabwe on the benefits of multi‐month PrEP dispensing to support PrEP continuation among female sex workers and was recommended recently by the World Health Organization as an approach to streamline PrEP delivery [20, 30, 31]. It also supports prior programmatic work from our PSUP team, which found that Kenyan clinics were moving towards efficiencies in PrEP refills with multi‐month PrEP dispensing and fast‐tracking stable PrEP clients through their visits even prior to the pandemic [25]. We heard about COVID‐related changes to laboratory procedures during PrEP initiation and refill visits that could be maintained after the pandemic. The pandemic resulted in a review of laboratory procedures in a number of clinics and resulted in streamlined PrEP delivery with fewer laboratory procedures. Finally, although only some clinics maintained task‐shifting approaches by the second interview, they allowed clinics to be flexible to the changing pandemic context and were also highly acceptable to clients because of faster clinic visits and having a network of providers to manage their care. Future studies could explore the effect of these modifications on PrEP uptake and continuation and ways to improve their acceptability among clinic staff and providers. The PrEP delivery modifications described here could also be included in PrEP guidance documents in Kenya and more broadly to promote efficient and client‐centred PrEP services.

The strengths of this study include the sample from two regions and multiple clinics in Kenya, longitudinal data collection and prior PrEP delivery experience among interviewees, which allowed us to achieve saturation of themes and observe changes in PrEP adaptations over time. However, the modifications described may not be generalizable to other populations and programmatic delivery settings in Kenya, particularly those not primarily focused on providing PrEP to HIV serodiscordant couples, as our study clinics were. There is a potential for selection bias with our qualitative sample, whereby clinic staff who were identified for participation in this study may have been those who were still actively engaged in PrEP delivery during the pandemic and were highly motivated to implement new approaches to support clients in continued PrEP use. We also only obtained data from one PrEP provider per clinic (without selecting providers based on length of employment at the clinic, cadre or other factors that could have influenced their views on PrEP delivery) and did not capture the perspectives of PrEP users, who may have felt differently about PrEP service delivery modifications during the COVID pandemic and could have provided additional insights on how these modifications supported their PrEP persistence. Moreover, cadres of PrEP providers were not equally represented and we did not include perspectives of other healthcare personnel involved in PrEP delivery, including pharmacists and community health workers. While our second interview was intended to take place after the COVID‐19 pandemic, it occurred during a time when COVID cases were stable but still occurring at moderate rates. Therefore, it is possible that some of the modifications that we noted as lasting through the second interview will be de‐implemented once case rates are further slow. We analysed data by time point of the interview (first interview or second interview) but did not explore themes by calendar time or trends in the COVID‐19 pandemic. Finally, we relied on participant self‐report of the PrEP delivery adaptations and we did not conduct clinic observations or review PrEP prescribing records to verify findings.

## 5 CONCLUSIONS

PrEP providers in Central and Western Kenya identified PrEP service delivery modifications—including adaptations to the clinic operations and staffing and procedures for PrEP demand creation, initiations, refills and retention—many of which were maintained beyond the initial stages of the COVID‐19 pandemic. Several modifications, including the use of partner notification services for demand creation and multi‐month PrEP refills, were maintained by the second interview, suggesting their acceptability among staff and clients. This work highlights opportunities for future PrEP programmes to potentially improve PrEP delivery through modifications to enhance efficiency, reduce stigma and provide person‐centred care, with the ultimate goal of supporting PrEP provision for clients during the COVID‐19 pandemic and beyond.

## COMPETING INTERESTS

JMB is an employee of Gilead Sciences. All other authors report no competing interests.

## AUTHORS’ CONTRIBUTIONS

JMB, EAB, NM, EMI, KN and KKM designed the parent study. JV, SDR and EJO conducted all analyses. JV wrote the manuscript. All authors reviewed and approved the final version of the manuscript.

JV: data analysis, results interpretation and manuscript first draft.

SDR: data analysis, results interpretation and edited manuscript.

EJO: data collection, data analysis, results interpretation and edited manuscript.

EMI, JFM, GO, KN and KKM: study design, results interpretation and edited manuscript.

AD, BK and NBT: data collection, results interpretation and edited manuscript.

NM, EAB and JMB: funding, study design, results interpretation and edited manuscript.

## FUNDING

This study was funded by the National Institute of Mental Health (NIMH) of the US National Institutes of Health (grant nos. R01MH095507 and R00MH118134) and the Bill & Melinda Gates Foundation (grant no. OPP10556051). JV was supported by the NIMH (grant no. K99 MH123369).

## DISCLAIMER

The content is solely the responsibility of the authors and does not necessarily represent the official views of the National Institutes of Health.

## Supporting information

COVID‐19 Key Informants Interview Question Guide

## Data Availability

The data that support the findings of this study are available from the corresponding author upon reasonable request.
