# Supplementary material for: Provider perspectives on service delivery modifications to maintain access to HIV pre‐exposure prophylaxis during the COVID‐19 pandemic: qualitative results from a PrEP implementation project in Kenya
Source: J Int AIDS Soc. 2023 Feb 5;26(2):e26055. doi: 10.1002/jia2.26055 (PMC9899492; doi:10.1002/jia2.26055)
Supplement: Supplementary file 1 — COVID‐19 Key Informants Interview Question Guide [file JIA2-26-e26055-s001.docx]

**COVID-19 Key Informants Interview Question Guide**

**Instructions for interviewer:**

1. Introduce yourself. Let the participant know why they were selected for the interview. Assure them that the information they share with you will be confidential and ensure that they have consented to the interview.
2. Primary interview questions are noted in **bold** text. As the interviewer, these are questions that must be asked or discussed with the participant. You don’t have to read the questions verbatim, but they are provided to ensure consistency across IDIs. The questions can be asked in any order depending on how the discussion with the participant is going, but by the end of the interview, all questions in bold should be asked.
3. Probing questions are indicated with a bullet. If a participant responds to the primary question with very little information, then these questions may be used to encourage further discussion. You do not have to cover all the probes provided, and you may not need to ask them if participant responds with enough information. Italic text highlighted in grey are instructions for interviewers. Such text should not be read aloud to the participant.

[Start recorder and read introduction]:

*********************************************************************************************

*Hi, my name is ____________. Thank you for agreeing to participate in an interview today. The COVID-19 outbreak has had a huge impact on healthcare workers and healthcare systems across the world and across Kenya and I understand this is a challenging time for all healthcare workers. I am interested in understanding your experiences as a healthcare worker during this time, how the healthcare system is supporting staff and clients, and how PrEP delivery has been impacted. I will ask you questions that you are free to answer in any way you wish. There are no wrong answers in this discussion. We are interested in knowing what you think, so please feel free to be open and share your point of view and elaborate on any of your points. If a question is unclear to you, please feel free to ask me to explain it.*

*It is very important that we hear your opinion. You do not have to answer all the questions. If you want to stop the discussion at any time, just let me know.*

**Topic 1: PrEP Delivery during COVID-19 Outbreak**

1. **What has been your experience delivering PrEP services at this clinic during the COVID-19 outbreak?**
   - *Remind participant of key themes they discussed during their first interview and ask them if there have been any changes recently.*
2. **What has changed with regard to PrEP delivery as compared to before the outbreak?**
   - What about as compared to the last time we spoke during the height of the COVID outbreak? *Remind participant of key themes they discussed during their first interview and ask them if there have been any changes recently in the following areas:*
     - How has the way this clinic is staffed changed?
     - Has the facility had any changes (e.g., changes in operating hours, temperature checks for clients, etc?)
     - If you think about the process that PrEP clients go through – from HIV testing to PrEP cousneling, prescribing, and dispensing – has anything changed about that process during this COVID outbreak?
     - How has the way the clinic conducts demand creation for PrEP changed?
     - Has the way in which clients are initiated on PrEP changed?
     - How has the way in which PrEP is delivered at follow up and refill visits changed? Have TCAs changed?
     - How have retention efforts changed?
3. **You’ve mentioned some changes in PrEP delivery due to the COVID-19 outbreak. How do you feel about those changes?**
   - How are the changes beneficial for PrEP delivery?
   - How do the changes hinder PrEP delivery?
   - Have the changes affected the PrEP delivery process in any way?
   - How confident are you that these changes to PrEP delivery will allow this clinic to continue PrEP delivery during this time?
   - Do you think any of the changes will remain after the COVID-19 outbreak? If so, which ones.
4. **Certain factors motivate people to use PrEP [maybe provide some examples here]. How has the outbreak impacted/affected/changed clients’ motivations to initiate or continue PrEP?** *Remind participant of key themes they discussed during their first interview and ask them if there have been any changes recently in the following areas:*
   - Have the reasons clients have for taking PrEP changed?
   - Have there been clients who have discontinued PrEP due to circumstances related to the outbreak?
   - Have there been clients who have re-started PrEP during the outbreak? If so, what were their motivations for doing so?

**Topic 2: Individual Preparedness and Concerns**

1. **What kind of training you have received related to COVID-19 since we last spoke in XXX?** *Remind participant of key themes they discussed during their first interview.*
   - Where and when did you receive this training?
   - Do you think this training has sufficiently prepared you for being able to talk with clients about COVID-19?
   - What are the most common questions or concerns you hear from clients about COVID-19?
2. **What measures has the hospital or clinic taken to protect staff during the COVID-19 outbreak since we last spoke in XXX?** *Remind participant of key themes they discussed during their first interview.* *The probes in this question are essential, please be sure they are answered.*
   - Do you feel these measures are adequate?
   - Do you feel supported by these measures? Do you feel supported by supervisors/other staff at the clinic? If yes, in what way do you feel supported?
   - What other things would you like to see the hospital or clinic do to protect staff?
   - What are specific resources you would like the clinic or hospital to provide (e.g., training, PPE, clinic infrastructure, mental health services, financial support/assistance)?
3. **What personal fears or concerns do you have about continuing to provide PrEP services during the outbreak?**
   - Do you feel safe providing services here? What makes you feel that way?
   - How have your feelings of fear or concern about continuing to provide PrEP services during the outbreak changed at all since we last spoke? *Remind participant of key themes they discussed during their first interview.*
4. **What fears or concerns do you have about how the outbreak will affect you personally?**
   - Do you have worries about job security or other economic impacts from the outbreak?
   - How have your worries about job security or other economic impacts from the outbreak changed at all since we last spoke? *Remind participant of key themes they discussed during their first interview.*
5. **How do the important people in your life (e.g., family, friend, other social support) feel about you continuing to provide services here? Do they have concerns about your safety?**
   - If yes, what are some of those concerns?
   - How have these concerns changed since we last spoke? *Remind participant of key themes they discussed during their first interview.*
6. **We know that the COVID-19 emergency has been a very stressful time for healthcare providers all over the world. What resources does this hospital or clinic have for staff to deal with fear or anxiety regarding contracting COVID-19?**

How have hospital/clinic staff supported each other during this time? *Be sure to probe participant to elicit detail here (e.g., “Can you describe a particular conversation with other staff members where you felt really supported?”, “How have you supported other hospital or clinic staff—what did you do to support them during this time?”)*

1. **What motivates you to continue providing PrEP services during the current outbreak?**
   - What makes you feel that way?
   - How have your motivations changed since we last spoke? *Remind participant of key themes they discussed during their first interview.*
2. **Is there anything else you would like to share related to your feelings of providing services during the current COVID-19 outbreak?**

**Thank you very much for continuing to provide PrEP services—provide thanks and encouragement to give participants some hope about the future.**
